# Atomic Force Microscopy of Side Wall and Septa Peptidoglycan From *Bacillus subtilis* Reveals an Architectural Remodeling During Growth

**DOI:** 10.3389/fmicb.2018.00620

**Published:** 2018-03-29

**Authors:** Kang Li, Xiao-Xue Yuan, He-Min Sun, Long-Sheng Zhao, Ruocong Tang, Zhi-Hua Chen, Qi-Long Qin, Xiu-Lan Chen, Yu-Zhong Zhang, Hai-Nan Su

**Affiliations:** ^1^State Key Laboratory of Microbial Technology, Marine Biotechnology Research Center, Shandong University, Jinan, China; ^2^Laboratory for Marine Biology and Biotechnology, Qingdao National Laboratory for Marine Science and Technology, Qingdao, China; ^3^College of Marine Life Sciences, Ocean University of China, Qingdao, China

**Keywords:** cell wall, peptidoglycan, structure, remodeling, atomic force microscopy

## Abstract

Peptidoglycan is the fundamental structural constituent of the bacterial cell wall. Despite many years of research, the architecture of peptidoglycan is still largely elusive. Here, we report the high-resolution architecture of peptidoglycan from the model Gram-positive bacterium *Bacillus subtilis*. We provide high-resolution evidence of peptidoglycan architecture remodeling at different growth stages. Side wall peptidoglycan from *B. subtilis* strain AS1.398 changed from an irregular architecture in exponential growth phase to an ordered cable-like architecture in stationary phase. Thickness of side wall peptidoglycan was found to be related with growth stages, with a slight increase after transition to stationary phase. Septal disks were synthesized progressively toward the center, while the surface features were less clear than those imaged with side walls. Compared with previous studies, our results revealed slight differences in architecture of peptidoglycan from different *B. subtilis* strains, expanding our knowledge about the architectural features of *B. subtilis* peptidoglycan.

## Introduction

Peptidoglycan is the major constituent of bacterial cell wall, and it is essential for bacteria to maintain their specific shape and to protect the cells from rupture by the internal turgor pressure (Typas et al., 2012). Moreover, peptidoglycan is important because it is the target of many antibiotics (Bugg et al., 2011). Elucidating the structure of peptidoglycan is a basic objective for microbiological research, but despite decades of work, the architecture of peptidoglycan is still not fully understood (Vollmer and Seligman, 2010; Turner et al., 2014). Although the chemical composition of peptidoglycan is well-characterized, the peptidoglycan architecture and their dynamics during growth and division are largely elusive. Several peptidoglycan models such as layered model and scaffold model have been proposed (Dmitriev et al., 2003; Gan et al., 2008). But direct observation of peptidoglycan architecture has been poorly documented so far.

Atomic force microscopy (AFM) is a powerful technique that allows direct observation off the surface structure of biological samples with high-resolution (Dufrêne, 2008, 2014; Müller and Dufrêne, 2011), and a series of surprising discoveries about the architecture of peptidoglycan from both isolated sacculi and living bacterial cells based on AFM works were reported during the past decade (Hayhurst et al., 2008; Andre et al., 2010; Turner et al., 2010, 2013; Wheeler et al., 2011; Dover et al., 2015). One of the breakthroughs was the first high-resolution architecture analysis of isolated sacculi from the rod-shaped bacterium *Bacillus subtilis* by direct observation with AFM in Hayhurst et al. (2008). The side wall peptidoglycan was reported to be organized into a regular structure of 50-nm wide “cables” with cross striations running across the short axis of the cells (Hayhurst et al., 2008). A coiled-coil model for peptidoglycan architecture was proposed based on AFM observations (Hayhurst et al., 2008). However, despite extensive researches, arguments about the peptidoglycan architecture remained. For example, studies with electron cryotomography suggested that glycan strands in Gram-positive cell walls run circumferentially around the cells (Beeby et al., 2013).

Peptidoglycan composition is known to change during growth. Both glycan chain length and crosslinkage are changing during the transition from exponential to stationary phase (Fordham and Gilvarg, 1974; Atrih et al., 1999; Typas et al., 2012). Bacteria can release D-amino acids into growth medium where they accumulate to millimolar concentrations in stationary phase (Lam et al., 2009). These D-amino acids can be incorporated into peptidoglycan and govern peptidoglycan remodeling in stationary phase (Lam et al., 2009). However, it remains unknown if peptidoglycan architecture changes depending on the growth phase.

Septa (or cross walls), which are formed between two bacterial daughter cells, are critical wall structures responsible for the bacterial division. A recent work showed that muropeptides with unprocessed stem peptides were accumulated in peptidoglycan at septa sites from *B. subtilis*, indicating a possible local difference in chemical composition between septa and side walls (Angeles et al., 2017). AFM studies on isolated sacculi pieces from *B. subtilis* showed that septal peptidoglycan was organized into ∼135-nm-wide “cable” like structures forming a spiral appearance toward the center (Hayhurst et al., 2008). However, no information on septal architecture in exponential and stationary phase is available.

In this report, peptidoglycan from *B. subtilis* strain AS1.398 was isolated and analyzed by high-resolution AFM. The results revealed the spatial organizations of side wall peptidoglycan and septa at a nanometer scale, suggesting the structural remodeling of the peptidoglycan during growth. Compared with previous studies, our results revealed slight structural differences in spatial organizations of peptidoglycan from different *B. subtilis* strains. This work expanded our current knowledge and provided new information about peptidoglycan architecture.

## Materials and Methods

### Bacterial Strain and Growth Condition

Bacterial growth was monitored by measuring the optical density at 600 nm (OD_600_) with a UV/VIS-550 spectrophotometer (Jasco, Japan). *B. subtilis* strain AS1.398 from a single colony was grown Luria-Bertani (LB) broth at 25°C overnight with shaking at 180 rpm. Then bacterial culture was diluted with fresh LB broth to a volume of 200 mL to reach a starting cell density of approximately 0.02 at OD_600_. The cell suspension was then incubated at 25°C with shaking at 180 rpm. The growth of the bacteria was monitored at OD_600_ at different time points, with three replicates at each time point. *B. subtilis* cells grown to mid-exponential phase (OD_600_≈1.2), late exponential phase (OD_600_≈1.8), and stationary phase were collected for optical microscopic imaging. Optical microscopic images were taken with an OMV optical microscope (Bruker AXS, Germany) affiliated with atomic force microscopy (Bruker AXS, Germany).

### Purification of Sacculi

According to growth curves, *B. subtilis* cells grown to mid-exponential phase, late exponential phase, and stationary phase were collected. Peptidoglycan was purified as described previously (Hayhurst et al., 2008). Briefly, cells were harvested, boiled (7 min), broken by ultrasonication (200∼400w) or high pressure cell disrupter (Constant Systems, Ltd., United Kingdom). When isolating intact sacculi, the breakage step was not needed. Extraction was treated by boiling in SDS (5% w/v), RNase (0.5 mg/ml), DNase (0.5 mg/ml), and pronase (2 mg/ml) treatment. Removal of accessory polymers was achieved by incubation in 48% v/v HF at 4°C for 24 h. Purified sacculi were washed at least three times with MilliQ water at room temperature. Then the samples were diluted in MilliQ water and air dried onto freshly cleaved mica before AFM imaging. At least three replicates were performed in each isolation experiment.

### AFM Operation

Atomic force microscopy imaging was carried out using a Multimode VIII AFM with Nanoscope V controller (Bruker AXS, Germany) equipped with an OMV optical microscope (Bruker AXS, Germany). All AFM imaging was carried out in scanasyst mode. Silicon cantilevers (XSC11/ALBS, MikroMash, Bulgaria) with a spring constant about 2.7 n/m were used for imaging in ambient conditions. Image processing and analysis were performed with AFM off-line software NanoScope Analysis (Bruker AXS, Germany).

### Statistical Analysis

Data are presented as the arithmetic mean ± standard deviation (SD). Statistical significance was evaluated using Student’s *t*-test. *p*-Values less than 0.05 were considered statistically significant.

## Results

### Growth Kinetics and Morphologies of Bacterial Cells

**FIGURE 1:**
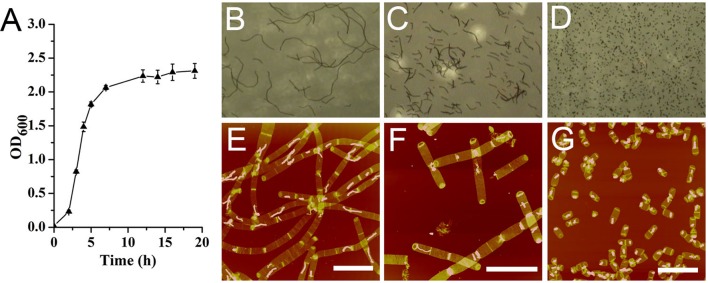
Growth kinetics of *Bacillus subtilis*. **(A)** Growth curve. Optical image of *B. subtilis* cells at mid-exponential phase **(B)**, late exponential phase **(C)**, and stationary phase **(D)**. Atomic force microscopy (AFM) images of sacculi isolated from *B. subtilis* cells at mid-exponential phase **(E)**, late exponential phase **(F)**, and stationary phase **(G)**. Scale bar: 10 μm.

*Bacillus subtilis* exhibited a typical bacterial growth curve (**Figure 1A**). *B. subtilis* cells formed long filamentous chains which could be as long as 150 μm in mid-exponential phase observed with optical microscope (**Figure 1B**). In late exponential phase, the filamentous chains of *B. subtilis* cells were much shorter than those in mid-exponential phase (**Figure 1C**). In stationary phase, only very short cells were observed (**Figure 1D**). Except for our *Bacillus* strain, some other *Bacillus* strains were known to be able to form long filamentous chains (Trick et al., 1984; Ajithkumar et al., 2002). It seemed that growth conditions could also influence the formation of filamentous chains in *Bacillus* species (Fan, 1970; Ferroni and Inniss, 1973).

From observations of isolated intact sacculi of the bacterial cells at different growth stages, multiple septation sites could be noticed along the bacterial filamentous chains (**Figures 1E,F**). Therefore it seemed that the filamentous chains of *B. subtilis* were not formed by loosely associated cells, but fast growing cells with multiple septa yet to be divided. The average cell length in stationary phase was 2.72 ± 0.63 μm (60 measurements from three replicates) (**Figure 1G**), while the measured average length between each adjacent septation sites was 3.78 ± 1.28 μm in mid exponential cells (70 measurements in 15 bacterial filamentous chains from three replicates), which was longer than the average cell length in stationary phase (*p* < 0.05).

### Structure of Side Wall Peptidoglycan

Thickness of isolated sacculi was measured in air by AFM. Samples from three independent replicates were used for measurements. The average thickness of single layered side wall peptidoglycan was 12.59 ± 0.89 nm (*n* = 59) in mid-exponential phase. This measured value was slightly larger than the measured thickness of side wall peptidoglycan in another *B. subtilis* strain in a previous report (Hayhurst et al., 2008), and the difference in the measured value might be due to the different strains used for experiments. The measured average thickness of single layered side wall peptidoglycan of our *B. subtilis* strain increased to 14.44 ± 0.92 nm (*n* = 52) in stationary phase, which was larger than that in mid-exponential phase (*p* < 0.05). This result indicates that the thickness of bacterial peptidoglycan might not be a fixed value, but varies during bacterial growth.

Broken sacculi from *B. subtilis* which exposed inner surface of the side wall peptidoglycan were imaged with AFM to check the surface features both the inner and outer surfaces. The inner surface of the side wall peptidoglycan exhibited a relatively rough surface feature, and the overall organization of inner-side peptidoglycan was largely parallel to the short axis of the cell (Supplementary Figure 1). However, the outer surface of sacculi was relatively featureless compared to the inner surface. It was suggested that hydrolysis of the peptidoglycan by endogenous autolysins might be one of the reasons that were responsible for such surface characteristics on outer side walls (Hayhurst et al., 2008). Our observations on the inner and outer side of the side wall peptidoglycan were in consistent with previous AFM observation (Hayhurst et al., 2008).

**FIGURE 2:**
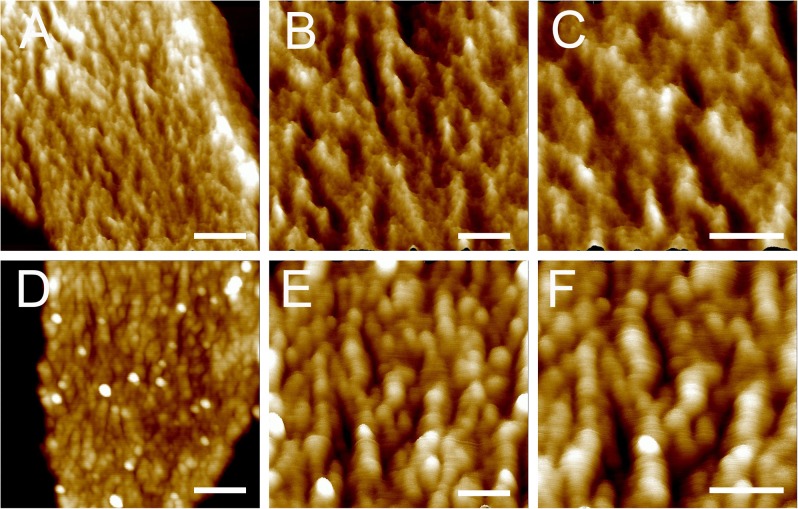
High-resolution AFM images of side wall peptidoglycan from *B. subtilis*. Peptidoglycan samples were from *B. subtilis* in mid-exponential phase **(A–C)**, and stationary phase **(D–F)**. Images were three-dimensional height images. Scale bar **(A,D)**: 200 nm; scale bar in other panels was 100 nm.

Next, the architecture of peptidoglycan on the inner surface of purified sacculi was imaged using high-resolution AFM. In stationary phase, the spatial organization of the peptidoglycan resembled the previously reported “cable-like” model (Hayhurst et al., 2008). These “cables” were densely packed together and roughly running in a parallel orientation (**Figures 2D–F**), and the result could be confirmed in each replicates. Small cables entangled into larger ones could sometimes be noticed. The average width of these “cables” was 29.11 ± 5.79 nm (*n* = 32), which was much smaller as compared to that in another *B. subtilis* strain in a previous report (Hayhurst et al., 2008). However, a structural difference was observed on side-wall peptidoglycans from *B. subtilis* in mid-exponential phase compared to that in stationary phase (**Figures 2A–C**). Peptidoglycan in mid-exponential phase seemed to be less ordered than in stationary phase, and it might be roughly characterized as a network like structure, with “ridge-and-groove” like appearances. These “ridge-and-groove” structures were largely parallel oriented. Small “ridges” were sometimes to be noticed to entangle into larger “ridges” (**Figures 2A–C**).

### Structures of Septal Peptidoglycan

**FIGURE 3:**
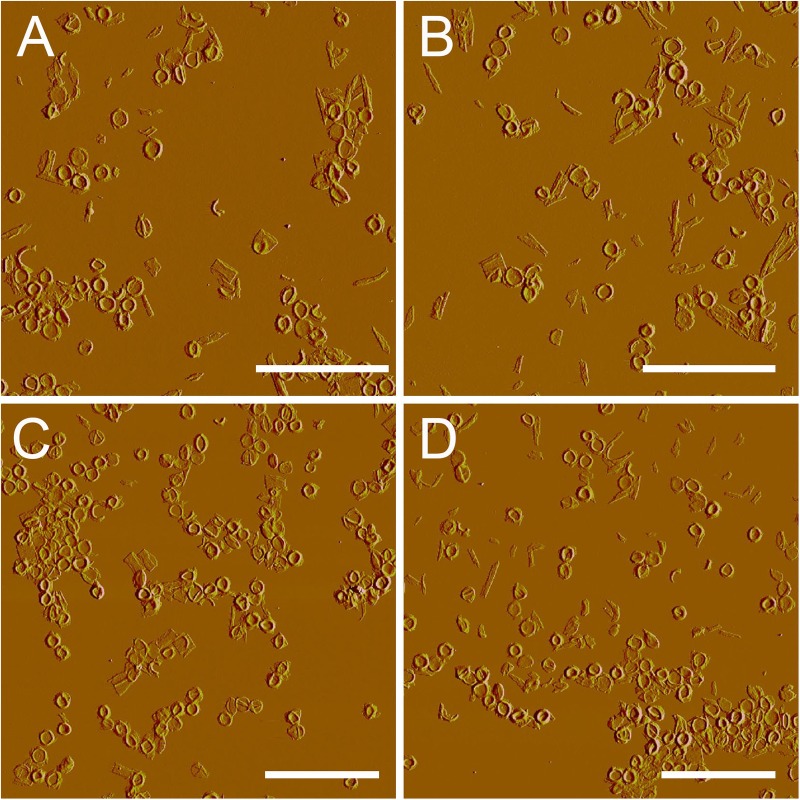
Large scale overview of isolated septa. After treatment with sonication (400w, three rounds), side wall peptidoglycan was severely broken, but septa remained largely intact. **(A–D)** Peak force error images. Scale bar: 10 μm.

**FIGURE 4:**
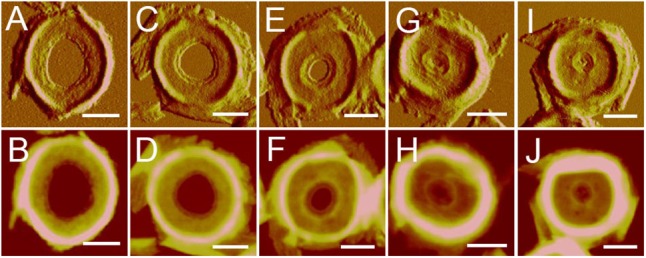
Progression of septa formation observed with AFM. Images showed the progression from early septa **(A,B)** to nearly-completed septa **(I,J)**. **(A,C,E,G,I)** Panels were peak force error images, and the **(B,D,F,H,J)** panels were corresponding height images. Scale bar: 500 nm.

After treatment with high-power sonication, the isolated sacculi were broken into pieces. It was surprising to notice that most of the side wall peptidoglycan was broken into small pieces, while large amounts of intact septa-like structures were observed (**Figure 3**). A large number of peptidoglycan fragments corresponded to incomplete septa, appearing as annulus-like structures. Annulus-like structures with attached side wall peptidoglycans could usually be observed (Supplementary Figure 2), further confirming that they were incomplete septa. When more septa were checked, septa structures that represent all stages of formation through the progression from newly forming septa to complete septa could be observed (**Figure 4**). A thin interior leading edge could be noticed in incomplete septal disk. Except the interior leading edge, thicknesses at other parts of the septal disks were evenly distributed. A likely process was that a thin leading ring was formed at the interior edge and then thickened with the growth of the septa, until the septa were completely sealed (**Figure 4**). This result may provide some new hints of how septa were progressively formed.

**FIGURE 5:**
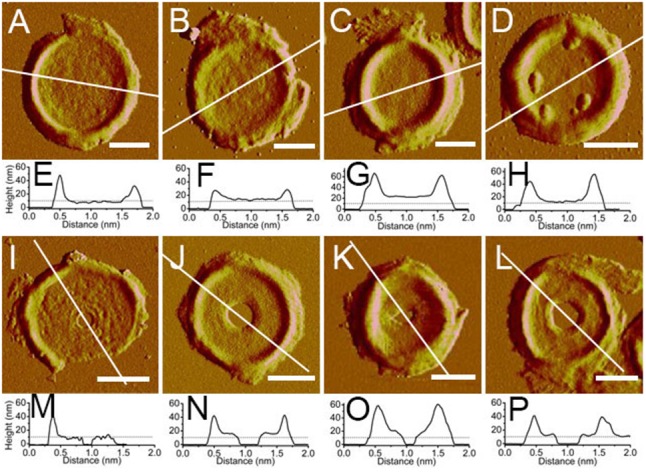
Atomic force microscopy images of isolated septa and their section analysis. **(A–D)** Complete septa; **(I–L)** incomplete septa; **(A–C,I–K)** were from *B. subtilis* in mid-exponential phase; **(D,I)** were from stationary phase; **(E–H,M–P)** were section analyses in their corresponding AFM images above. All images were peak force error images. Section analyses were performed in their corresponding height images. Lines indicated the position for section analyses. Scale bar: 500 nm.

The thicknesses of complete and incomplete septa from cells at mid-exponential phase (*n* = 48) and stationary phase (*n* = 45) were measured. The thicknesses of most septa at both growth stages were between 11 and 16 nm. However, in exponential phase, small amount of septa with unusual thicknesses of less than 8 nm (5 out of 48) or as much as 20 nm (4 out of 48) could be found (**Figure 5**). Septa with different thicknesses could be noticed not only in complete septa but also in incomplete septa, indicating that formation of different thicknesses was determined before the septa were complete. Unlike the possible spiral cable-like structure of the septa in previous report (Hayhurst et al., 2008), the surfaces of most septal disks isolated from our *B. subtilis* strain were relatively smooth, with no obvious supramolecular structural organizations as in side walls. However, only in some case, “cables” like organizations with ∼35 nm (*n* = 7) width forming concentric rings toward the center were observed on septa with thin thickness (∼8 nm) (Supplementary Figure 3). The width of the “cables” in our strain was much smaller than that in the other strain in previous report (Hayhurst et al., 2008).

## Discussion

Bacterial peptidoglycan plays important roles in various biological processes (de Pedro and Cava, 2015). The peptidoglycan of Gram-positive bacteria is significantly thicker and more complex than the peptidoglycan of Gram-negative bacteria, and the three dimensional architectures of Gram-positive peptidoglycan was largely unclear in the past. Application of novel high-resolution techniques such as AFM in recent years revealed the surface architecture of bacterial peptidoglycan (Turner et al., 2014). To date, the only previous AFM work to study the architecture of isolated sacculi from model Gram-positive rod bacterium *B. subtilis* was reported by Hayhurst et al. (2008). They found that the peptidoglycan in the inner surface of the sacculi was organized into a regular structure of 50-nm wide “cables” (Hayhurst et al., 2008). We found that the inner surface of side wall peptidoglycan from *B. subtilis* in stationary phase exhibited a “cable” like structure, quite similar to the previous observation (Hayhurst et al., 2008). However, the average width of the “cables” was about 29 nm, which was much smaller than that in the other *B. subtilis* strain in previous report (Hayhurst et al., 2008). This result suggests the existence of differences in peptidoglycan architectures from different *Bacillus* strains.

A coiled-coil model was proposed based on previous AFM observations about the side wall peptidoglycan from *B. subtilis* (Hayhurst et al., 2008). However, this model was argued because the peptidoglycan from *B. subtilis* seemed to be a uniformly dense layer observed with electron cryo-tomography (Beeby et al., 2013) or electron cryo-microscopy (Matias and Beveridge, 2005). Our work confirmed that the “cable” like structure in side wall peptidoglycan from *B. subtilis* exists. Work is still needed in the future to reconcile the observations by different techniques.

Our high-resolution AFM images showed that there were slight differences in architecture of side wall peptidoglycan from different growth stages. In mid-exponential phase, the side wall peptidoglycan was organized into a “ridge-and-groove” like structure, which differed from the “cable” like structure in side wall peptidoglycan from stationary phase. Moreover, the thicknesses of side-wall peptidoglycan slightly increased from exponential phase to stationary phase. This variation in thicknesses might be related to the architectural changes in side walls. It has been known that bacterial peptidoglycan undergoes a remodeling process during different growth stage, both in Gram-positive and Gram-negative bacteria (Typas et al., 2012). In stationary phase, the crosslinks in peptidoglycan from *B. subtilis* were found to increase (Atrih et al., 1999). The peptidoglycan remodeling in bacteria at stationary phase was known to be governed by D-amino acids (Lam et al., 2009). Our work on the architecture of peptidoglycan from *B. subtilis* at different growth stages might reflect a remodeling of spatial organization in peptidoglycan structures.

The septum is an important structure that is responsible for the division in Gram-positive bacteria (Wu and Errington, 2011). In our work, we found that when treated with high-power sonication, side wall peptidoglycan was mostly broken into small pieces, leaving large amounts of intact septa. Previous research indicated that the circumferential stress in bacterial cells was greater than the longitudinal stress (Yao et al., 1999), and therefore it is likely that the general mechanics of stress in rod shaped cells are the possible reason for the side wall splitting while the septa remains largely intact. Another possible explanation that could not be fully excluded was that there might be difference in rigidity between side walls and septa. A recent work showed that local differences in the chemical composition of peptidoglycan between septa and side walls existed in *B. subtilis* (Angeles et al., 2017). However, whether local differences in chemical composition would result in different rigidity, or whether the septa might be truly more rigid than side wall peptidoglycan is unknown and awaits further analysis.

Previous AFM work suggested that the septal disk had up to three cables across their radius forming a spiral like structure toward the center (Hayhurst et al., 2008). However, results with the *B. subtilis* strain in this report showed that the septal disk of most observed septa were relatively smooth, with no obvious surface features. Apart from the surface features of septa, there were slight differences in the thicknesses and spatial organization of side wall peptidoglycan between the *B. subtilis* strain in previous report (Hayhurst et al., 2008) and the strain we used. Therefore, we consider that the differences in the surface features of septal disks might result from different bacterial strains used.

## Author Contributions

H-NS, Y-ZZ, and X-LC conceived and designed the experiments. KL, X-XY, H-MS, L-SZ, RT, Z-HC, and H-NS performed the experiments. H-NS and Q-LQ analyzed the data. H-NS wrote the paper. All authors read and approved the finalized manuscript.

## Conflict of Interest Statement

The authors declare that the research was conducted in the absence of any commercial or financial relationships that could be construed as a potential conflict of interest.
