# Supplementary material for: Atomic Force Microscopy of Side Wall and Septa Peptidoglycan From Bacillus subtilis Reveals an Architectural Remodeling During Growth
Source: Front Microbiol. 2018 Mar 29;9:620. doi: 10.3389/fmicb.2018.00620 (PMC5884923; doi:10.3389/fmicb.2018.00620)
Supplement: Supplementary file 1 [file Image_1.PDF]

Supplementary Material

Atomic Force Microscopy Research on Side Wall and Septa Peptidoglycan from *Bacillus subtilis* Reveals an Architectural Remodeling during Growth

Kang Li[1], Xiao-Xue Yuan[1], He-Min Sun[1], Long-Sheng Zhao[1], Ruocong Tang[1], Zhi-Hua Chen[1], Qi-Long Qin[1], Xiu-Lan Chen[1], Yu-Zhong Zhang[1,2,3], Hai-Nan Su[1,3,]*

[1]State Key Laboratory of Microbial Technology, Marine Biotechnology Research Center, Shandong University, Jinan, China; [2]Laboratory for Marine Biology and Biotechnology, Qingdao National Laboratory for Marine Science and Technology, Qingdao, China; [3]College of Marine Life Science, Ocean University of China, Qingdao, China

*** Correspondence:** Hai-Nan Su: suhn@sdu.edu.cn

1 Supplementary Figures

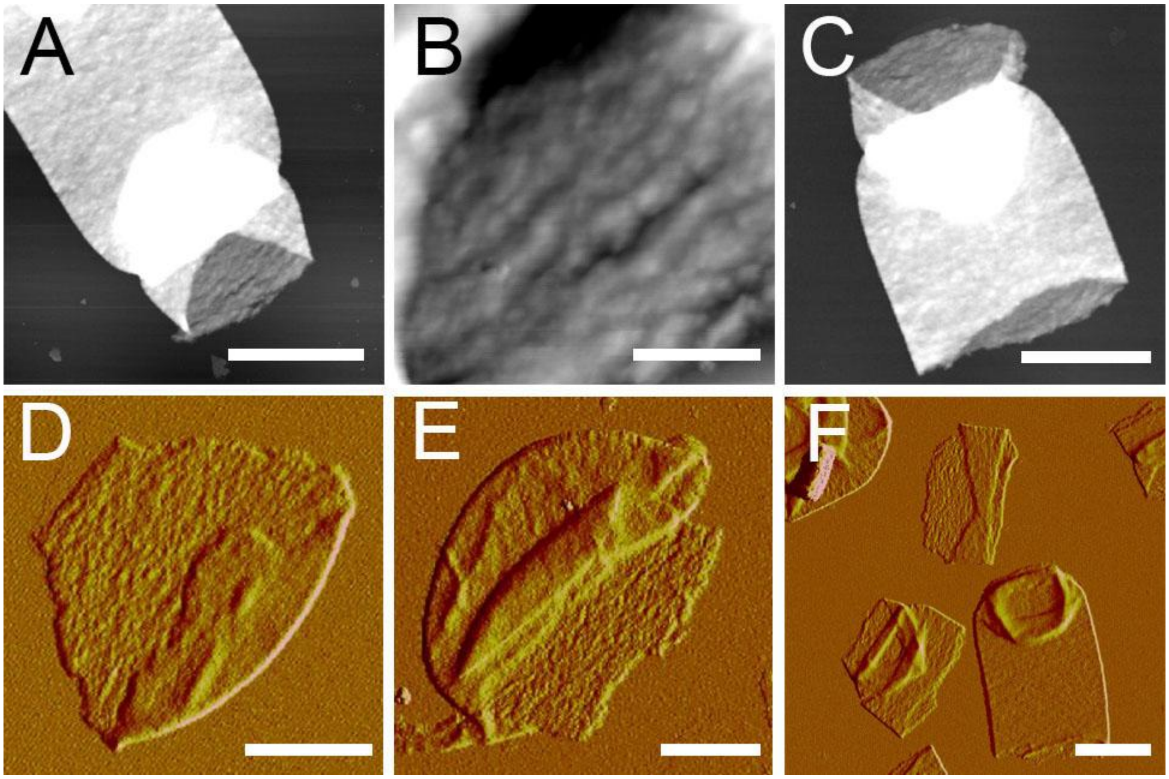

Supplementary Figure 1. AFM images of broken sacculi of *B. subtilis*. Broken sacculi indicating the different surface features between outer surface and inner surface. B was enlarged from A. A-C,

height images. E-F, peak force error images. Scale bar: A, 1 μm; B, 200 nm; C, 1 μm; D, 0.5μm; E, 0.5μm; F, 1μm.

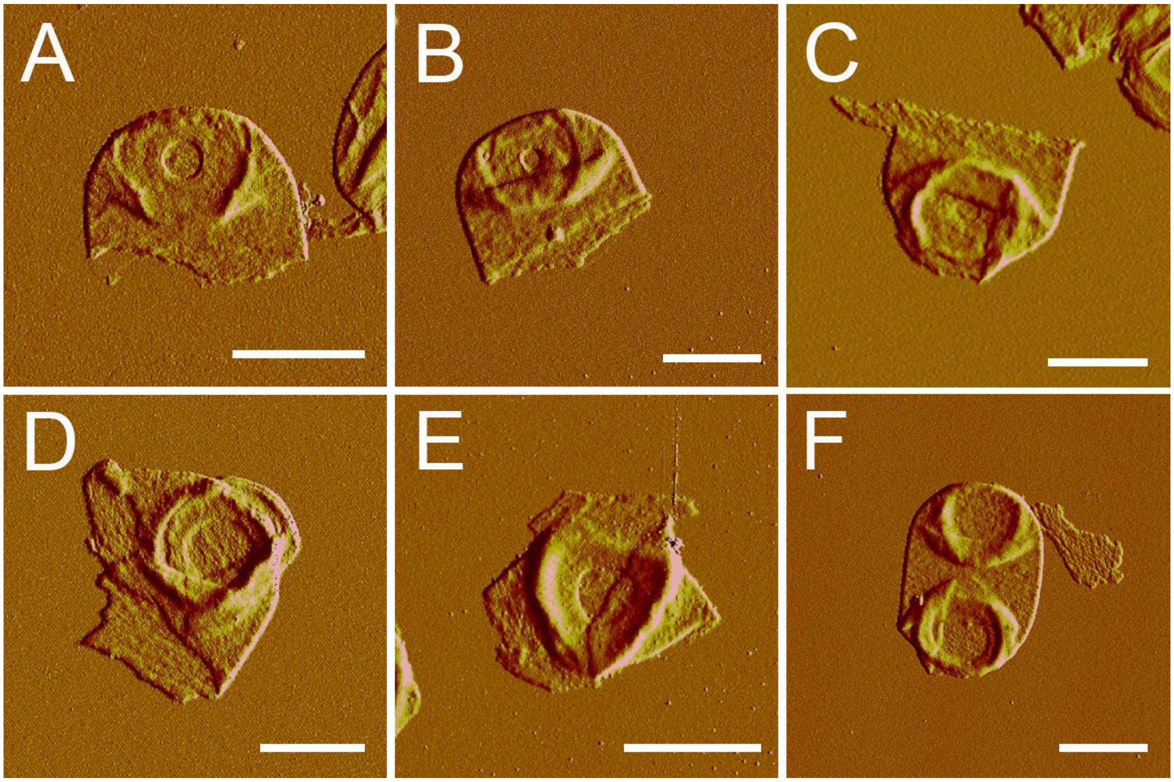

Supplementary Figure 2. Incomplete septa attaching side wall peptidoglycan. All images were peak force error images. Scale bar, 1 μm.

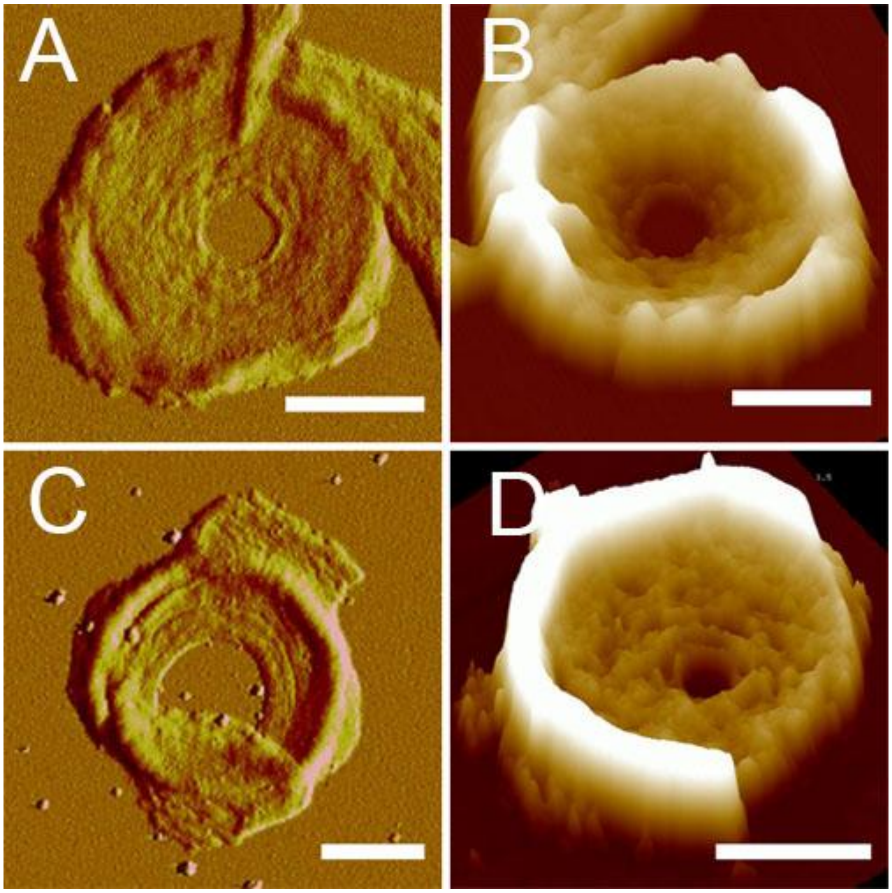

Supplementary Figure 3. Incomplete septa indicating the architecture of septal disk. A, C, peak force error images. B, D, three dimensional height image of A and Fig. 5I, respectively. Scale bar, 0.5 μm.
